# The expression of miRNA-152-3p and miRNA-185 in tumor tissues versus margin tissues of patients with chemo-treated breast cancer

**DOI:** 10.1186/s13104-021-05647-z

**Published:** 2021-06-16

**Authors:** Asma Safi, Soheila Delgir, Khandan Ilkhani, Azam Samei, Seyyed Reza Mousavi, Zahra Zeynali-Khasraghi, Milad Bastami, Mohammad Reza Alivand

**Affiliations:** 1 Clinical Research Development Unit, Shohada Hospital, Tabriz University of Medical Sciences, Tabriz, Iran; 2 Department of Medical Genetics, Faculty of Medicine, Tabriz University of Medical Sciences, Tabriz, Iran; 3 Department of Laboratory Sciences, School of Medical Sciences, Kashan University of Medical Sciences, Kashan, Iran

**Keywords:** Breast cancer, Chemoresistance, microRNAs, miR-152, PI3K/AKT pathway, Drug resistance

## Abstract

**Objective:**

Breast cancer (BC) is the most significant and lethal type of cancer in women. Although there are many newly develop chemotherapy drugs for patients with BC treating at various stages, drug resistance is the most important obstacle in their effectiveness for BC treatment. On the other hand, microRNAs are considered key regulators of genes involved in carcinogenesis and chemoresistance in cancers. The purpose of this study was to evaluate the role of miR-152-3p and miR-185 in intrinsic chemoresistance and proliferation of BC. In addition, the potential role of these miRNAs during chemoresistance was evaluated through possible signaling pathways.

**Results:**

Here, miR-152-3p was significantly downregulated in tumor tissues compared to the corresponding margin tissues in patients with BC (*p*-value ≥ 0.04407 and fold change = − 2.0552). In contrast, no statistically significant difference was observed in the miR-185 expression between the two groups. Furthermore, no significant correlation was found between the expression of these two miRNAs and subfactors, including cancer family history, abortion, and age. Downregulation of miR-152-3p could be considered a promising regulator of BC chemoresistance.

**Supplementary Information:**

The online version contains supplementary material available at 10.1186/s13104-021-05647-z.

## Introduction

Breast cancer (BC) is the most common malignancy among women worldwide and the main cause of cancer-related mortality [1–3]. According to the world health organization (WHO) reports, the incidence rates are approximately 1.7 million new cases and more than 520,000 annual deaths [4].

During the past few decades, new treatment approaches for BC have significantly developed, which not only decreased the mortality rate but also improved patients’ quality of life [5–7]. Recent studies revealed several intricate mechanisms that are involved in intrinsic and acquired chemoresistance [8–10]. There are two forms of drug resistance in cancer: intrinsic resistance and acquired resistance. Intrinsic resistance refers to resistance prior to prescribing the drug, and this is due to the innate ability of cancer cells to survive in drug-related clinical concentrations. Poor initial response to treatment indicates that a patient has intrinsic drug resistance. According to Verheul and Pinedo, more than 50% of all cancer patients are resistant to chemotherapy before starting treatment (intrinsic resistance), and other patients also resist during treatment (acquired resistance) [11]. Intrinsic and acquired resistance to chemotherapeutic agents is considered the main obstacle to successful treatment in patients with BC [12]. Thus, investigation of the molecular mechanisms of drug resistance in BC may help reach better-targeted treatment by significantly improving therapeutic outcomes and prognosis for patients with chemo-resistant BC.

Increasing evidence shows that aberrant activation of the phosphatidylinositol-3-kinase (PI3K)/AKT signaling pathway is common in various types of neoplasms. Furthermore, PTEN plays a negative regulatory role in the PI3K/AKT signaling. It is involved in many important processes such as cell growth and apoptosis and drug resistance/sensitivity. Thus, targeting PI3K/AKT and its downstream pathways may provide new hope for therapeutic interventions of cancers [13–16]. MicroRNAs (miRNAs) are a family of noncoding-RNAs, playing a crucial role in post-transcriptionally gene expression modulation [17]. Recently, it has been determined that miRNAs can play key roles during cancer development and resistance to chemotherapy by acting as oncogenes or tumor suppressors [18–20].

A bulk of evidence indicated that both miR-152-3p and miR-185 as tumor suppressors and induced progression of multidrug resistance in different human tumors, including osteosarcoma [21], epithelial ovarian cancer [22], colorectal [23], liver cancer stem cells[24], NSCLC cells [25, 26], gastric [27], melanoma [28], nasopharyngeal carcinoma [29], and BC [26]. Hence, the association of miR-152-3p and miR-185 expression levels with chemoresistance could help better understanding the molecular and biological processes involved in response to chemotherapy in various types of cancers, especially BC.

In the current study, the expression level of miR-152-3p and miR-185 was evaluated in tumor and margin tissues of patients with BC. Moreover, in combination with bioinformatics analysis, the potential role of these miRNAs during chemoresistance was evaluated through possible signaling pathways.

## Main text

### Material and methods

#### Tissue specimens

Two hundred tumor tissues and corresponding normal margin of tumor tissues were collected from patients with BC from 2018 to 2019 who underwent surgery at Noor Nejat Hospital, Tabriz, Iran. The collected tissues were dissected by a pathologist for diagnosing BC foci as well as margin tissues. After chemotherapy courses, it was determined- by the oncologist- that eighty tumor tissues have shown initial resistance to chemotherapy. This study was approved by the Ethics Committee of Clinical Research of Tabriz University of Medical Sciences (Ethical code: IR.TBZMED.REC.1397.1048). Demographic and clinical characteristics were collected from the patients' records (Additional file 1: 1). The specimens were obtained from mastectomy immediately frozen and stored at − 80 °C before RNA isolation.

### RNA isolation

Tissue samples were cut in 20 μm-thick sections then were homogenized in liquid nitrogen. Each of the samples was directly immersed in TRIzol reagent (GeneAll, South Korea). The purity of extracted total RNAs was evaluated by the NanoDrop spectrometer (Thermo Scientific, USA) and gel electrophoresis. Finally, isolated RNAs were eluted in 30 μL of RNase-free water and stored at − 80 °C until use.

### cDNA synthesis and Real-time PCR

Complementary DNA (cDNA) was synthesized using reverse-transcriptase enzyme (Thermo Fisher, US), buffer (Thermo Fisher, US), dNTP, and miRNA- specific stem-loop-primers (Sinacolon Co. Iran, Tehran) for miR-185-5p and miR-152-3p. The U6 snRNA was also considered the reference gene. This mix of cDNA synthesis was carried out by BIO-RAD-Gradient thermocycler PCR (Germany) at the temperature program of 30 min at 16 °C, 30 min at 42 °C and the last step to inactivate the enzyme the reaction was incubated at 5 min at 75 °C. Ampliqon SYBR® Green master mix (Denmark) and miR-185-5p, miR-152-5p, and U6 specific forward primers and common reverse primers were used for quantitative Real-time PCR (qRT-PCR). All qPCR reactions were performed in triplicate. Real-time-PCR was done by LightCycler® 96 Real-time PCR Cycler (Roche) system, in two steps as follows: for miR-185-5p: 10 min at 94 °C, 40 cycles of 15 s at 94 °C and 30 s at 54 °C. For miR-152-5p: 10 min at 94 °C, 40 cycles of 15 s at 94 °C, and 30 s at 60 °C. Then, for RNU6 amplification:10 min at 94 °C, 40 cycles in 15 s at 94 °C and 20 s at 56 °C. The relative expression levels of miRNAs were calculated using the 2^−ΔΔCt^ method relative to U6. The results were shown by LightCycler® 96 v1.1.0 Software. The primer sequences are shown in Table 1.

**Table 1:** Stem-loop primer (cDNA synthesis) and real-time primer sequences

Micro RNAs	Primer sequences
miR-185-5p SLP*	5'-GTCGTATCCAGTGCAGGGTCCGAGGTATTCGCACTGGATACGACTCAGGA-3'
miR-152-3p SLP	5'-GTCGTATCCAGTGCAGGGTCCGAGGTATTCGCACTGGATACGACCCAAGTT-3'
U6 SLP	5'-GTCGTATCCAGTGCAGGGTCCGAGGTATTCGCACTGGATACGACAAAAATAT-3'
miR-185-5p Forward	5'-GGAGAGAAAGGCAGTTCCTGAC-3'
miR-152-3p Forward	5'-CCCTCAGTGCATGACAGAACTTG-3'
Universal reverse primer for miR-152-3p and miR-185-5p	5'-GTGCAGGGTCCGAGGT-3'
U6 Forward	5'-GCTTCGGCAGCACATATACTAAAAT-3'
U6 Reverse	5'-CGCTTCACGAATTTGCGTGTCAT-3'

*SLP stem-loop

### Statistical analysis

#### Data analysis

Shapiro–Wilk test was performed to assess the normality of data. Next, the gene expression differences were compared between tumor tissues and margins using paired t-test and delta-CTs. Two-sample paired t-test was performed to compare the miRNA expression levels with subsets of the study, including cancer family history, abortion history, and age. The gene expression levels in subsets of the study, including abortion history, age, and cancer family were compared. A t-test for the logarithm2 of fold changes was applied to compare gene expression levels among subsets of the study. Statistical analyses were done by R software version 0.5.1, and *P*-value of ≤ 0.05 was considered statistically significant. The results were presented as mean ± standard error of mean (SEM).

#### In silico analysis of miR-152-3p targets and related molecular signaling pathways

To validate targetome of miR-152-3p, miRTarBase 7.0 [29] database was applied. Further, in silico analysis was employed to achieve the vision of biological procedures that are controlled by miR-152. All target genes of miR-152-3p experimentally verified were extracted from the miRTarBase 7.0 database. Finally, to enrich related signaling pathways to miR-152-3p dysregulation, target genes were investigated in the WEB-based Gene Set Analysis Toolkit (webgestalt) database (http://www.webgestalt.org/), and the Over-Representation enrichment (ORA) was selected and pathways were enriched based on Reactome database. The reference gene list was set to "genome-protein-coding," and the adjustment p-value was set as Benjamini-Hochberg. Other parameters were set as defaults.

## Results

### MiR-152-5p and miR-185-5p expression levels

**Fig. 1:**
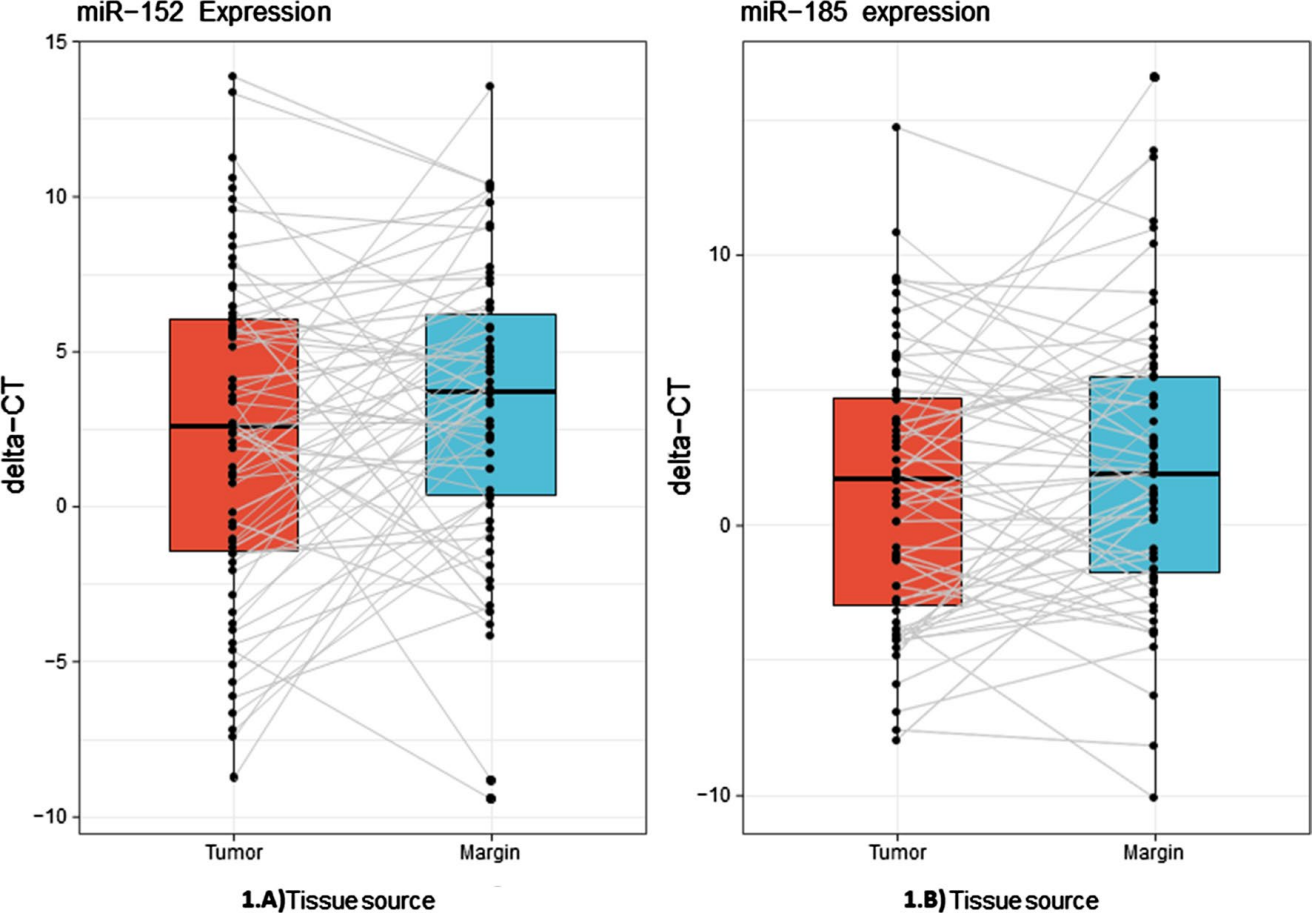
The expression of miRNAs in tumor tissues and corresponding margin tissues. **A** The miR-152-3p expression in tumor tissues and corresponding margins. **B** The miR-185-5p expression in tumor tissues and corresponding margins. In both figures, ΔCTs were used to show the expression level normalized to U6 snRNA in tumor and normal tissues

Paired T-test results revealed that the miR-152-3p expression level decreased in tumor tissues compared to the corresponding margins (*p*-value ≥ 0.04407 and fold change = -2.0552) (Fig. 1A). On the contrary to miR-152-3p, no statistically significant change was observed in the miR-185-5p expression levels between tumor tissues and the corresponding margins (*p*-value ≤ 0.1017 and fold change = -1.6621) (Fig. 1B).

### Subgroup analysis

The results of two-sample t-test demonstrated that firstly, there was no significant different expression levels of miR-152-5p and miR-185-5p among patients with and without abortion history in the past (p-value ≤ 0.4864, fold change = 0.70113) (Additional file 1: 2.a), (*p*-value ≤ 0.6572, fold change = 0.44646) (Additional file 1: 3.a). Moreover, there was no significant difference in the expression level of miR-152-5p (*p*-value ≤ 0.2623) (fold change = -1.1361) (Additional file 1: 2.b) and miR-185-5p (*p*-value ≤ 0.9613, fold change = 0.048805) between patients ≤ 50 and > 50 ages, respectively (Additional file 1: 3.b). However, there was no significant difference in the expression level of miR-152-5p between patients with and without cancer family history (Cancer family history means that a close relative has BC) (*p*-value ≤ 0.09579, fold change = -1.6964) (Additional file 1: 2.c). The miR-185-5p expression level was significantly different between the patients with and without cancer family history (*p*-value ≤ 0.02586, fold change = -2.293) (Additional file 1: 3.c).

### In silico analysis

The 158 experimentally validated gene targets of miR-152-3p were obtained by the miRTarBase 7.0 database. The miRNA-gene interaction was depicted by Cytoscape V 3.7.1 (Additional file 1: 4). ORA database revealed that ten categories of the biological processes were enriched among miR-152-3p target genes. All 10 of these biological processes are shown in (Additional file 1: 5). Moreover, the ORA analysis discovered ten significant signaling pathways in which the targets of miR-152 are involved (Table 2).

**Table 2:** Pathways that are enriched among target genes of miRNA-152-3p

Gene set	Pathway name	FDR	*P*-value	Fold enrichment detail
hsa05215	Prostate cancer	5.9238E−6	2.8840E−9	O = 11; E = 0.96; R = 11.37
R-HSA-8943723	Regulation of PTEN mRNA translation	8.1607E−6	1.1011E−8	O = 5; E = 0.08; R = 55.72
hsa04151	PI3K-Akt signaling pathway	8.1607E−6	1.1919E−8	O = 18; E = 3.52; R = 5.10
hsa05206	MicroRNAs in cancer	1.5061E−5	2.9330E−8	O = 12; E = 1.49; R = 8.02
hsa04550	Signaling pathways regulating pluripotency of stem cells	5.2459E−5	1.2770E−7	O = 11; E = 1.38; R = 7.93
hsa05224	Breast cancer	6.8102E−5	2.2617E−7	O = 11; E = 1.46; R = 7.50
hsa05165	Human papillomavirus infection	6.8102E−5	2.3209E−7	O = 16; E = 3.37; R = 4.73
R-HSA-426496	Post-transcriptional silencing by small RNAs	8.2303E−5	3.2056E−7	O = 4; E = 0.06; R = 57.32
hsa05214	Glioma	1.0421E−4	4.8141E−7	O = 8; E = 0.70; R = 11.30
R-HSA-8934593	Regulation of RUNX1 Expression and Activity	1.0421E−4	5.0736E−7	O = 5; E = 0.16; R = 29.50

*E* number of expected gene in the category, *FDR* false discovery rate, *O* number of observed target genes in the category, *R* fold enrichment

## Discussion

Therapeutic resistance to chemotherapy is considered the most important global health challenge for patients with cancers, especially patients with BC [6, 30, 31]. Recently, numerous experimental and clinical studies have shown that miRNAs participate in several cellular events, particularly in resistance/sensitivity to drugs and cancer progression [32, 33]. Therefore, identifying drug resistance-specific miRNAs and their target genes is critical for understanding their role in BC chemotherapy. Further, they could be potential candidates for therapeutic intervention [34, 35].

In the current study, based on previous studies and in silico analysis, it was predicted that miR-152-3p and miR-185 could be involved in BC chemoresistance. For this reason, we evaluate and compare the expression of the aforementioned miRNAs in tumor tissues relative to the margins from BC-resistant patients to chemotherapy. As mentioned in the results, we found that the expression level of miR-152-3p was significantly downregulated in BC tumor tissues compared to margin tissues. According to the previous studies, concerning chemoresistance and proliferation in various types of cancers, it has been shown that there is a significant relationship between miR-152-3p with chemoresistance and proliferation. For example, A Maimaitiming et al. proved the tumor-suppressive role of miR-152 by significantly downregulation in BC tissues relative to paired adjacent noncancerous tissues. Besides, he found that miR-152 overexpression significantly inhibited proliferation, migration, and invasion of BC cells [36].

Wen, Y.-Y et al. reported that miR-152 downregulation is associated with BC development. Moreover, induction of miR-152 could sensitize BC cells to the paclitaxel therapy by targeting β-catenin and PKM2 repression [37]. Shuke Ge et al. found that miR-152 plays a tumor-suppressive role in BC through negative regulation of PIK3CA expression and AKT and RPS6 inhibition, which leads to suppression of BC cell proliferation [38].

Moreover, Xu Chen et al. [26] found that the miR-148/152 family attenuates Adriamycin resistance of BC cells and tissues by downregulating the SPIN1, which is a protein highly expressed in human cancers, especially BC. [39, 40]. Therefore, downregulation of miR-152-3p in tumors relative to adjacent tissues may result in an elevated expression level of SPIN1 and resistance to chemotherapy.

According to bioinformatics results, among important molecular pathways enriched by the webgestalt database, PI3K-Akt signaling pathway and regulation of PTEN mRNA translation are important enriched signaling pathways in which the targets of miR-152-3p are involved, and there may be a relationship between miR-152-3p expression and BC chemoresistance. The PI3K-Akt signaling is an intracellular signaling pathway involved in pivotal processes such as proliferation, cell survival, and angiogenesis [41]. PTEN acts as a tumor-suppressor gene and negatively regulates PI3K-Akt signaling [42]. In the upstream of Akt, PTEN blocks the formation of phosphatidylinositol-3, 4, 5-trisphosphate (PIP3) from phosphatidylinositol-4, 5-bisphosphate (PIP2), thereby inhibiting PI3 kinase (PI3K) activity. Several human tumor cell lines have been reported to evade apoptosis through the excessive activation of the PI3K/Akt pathway, which is the result of a mutation or downregulation of PTEN [43]. Additionally, it has been demonstrated that constitutive activation of PI3K-Akt signaling causes cell resistance to many chemotherapy agents by promoting proliferation and inhibiting apoptosis of cancer cells [44]. M Alam et al. showed the significant loss of PTEN expression in 26.4% BC cases, suggesting loss of PTEN expression could play a key role in breast carcinogenesis, due to lack of control of the signaling pathways such as likely PI3K-Akt signaling that mediates cellular processes like apoptosis and migration [45].

Finally, demographic characteristics of the current study showed that no significant difference was seen between the miR-152 and miR-185 expression levels. Some demographic factors such as cancer, family history, or abortion history need further studies to be clarified.

## Conclusion

The findings of the current study suggested miR-152-3p as a potential biomarker in BC chemoresistance patients. Besides, the PI3K/Akt signaling was predicted as a possible key modulator during BC chemoresistance. Further studies are needed to elucidate these findings.

## Limitations

The small sample size was considered the limitation of this study. Also, applying only one miRNA-target database could be mentioned as another limitation.

## Supplementary Information

**Additional file 1. 1.** Demographic characteristics of the breast cancer patients. **2.** The correlation of miR-152-3p expression level with subgroup analysis. **a** The expression of miR-152-3p in patients with and without abortion history. **b** The miR-152-3p expression in patients ≤50 and >50 ages. **c** The miR-152-3p expression in patients with and without cancer family history. In both figures, LFC was used to show the expression level was normalized to U6 snRNA in the two groups. LFC means base the logarithm2 of fold change. **3.** The correlation of miR-185-5p expression level with subgroup analysis. **a** The miR-185-5p expression in patients with and without abortion history. **b** The expression of miR-185-5p in patients ≤50 and >50 ages. **c** The miR-185-5p expression in patients with and without cancer family history. In both figures, LFC was used to show the expression level normalized to U6 snRNA in the two groups. LFC means base the logarithm2 of fold change. **4.** The miR-152 target interaction depicted by Cytoscape 3.7. **5.** Top 10 enriched biological processes among target genes of miR-152-3p. *GO* Gene Ontology, *FDR* false discovery rate.

## Data Availability

The data that support the findings of this study are available on request from the corresponding author. The data are not publicly available due to privacy or ethical restrictions.

## Abbreviations

BC: Breast Cancer
MicroRNA: MiRNA
HCC: Hepatocellular carcinoma
NSCLC: Non-small cell lung cancer
PI3K/AKT: Phosphatidylinositol-3- kinase
SPIN1: Spindlin1
MCL: Mantle cell lymphoma
PTEN: Phosphatase and tensin homolog
