# Supplementary material for: The expression of miRNA-152-3p and miRNA-185 in tumor tissues versus margin tissues of patients with chemo-treated breast cancer
Source: BMC Res Notes. 2021 Jun 16;14:234. doi: 10.1186/s13104-021-05647-z (PMC8207775; doi:10.1186/s13104-021-05647-z)
Supplement: Supplementary file 1 — Additional file 1. 1. Demographic characteristics of the breast cancer patients. 2. The correlation of miR-152-3p expression level with subgroup analysis. a The expression of miR-152-3p in patients with and without abortion history. b The miR-152-3p expression in patients ≤50 and >50 ages. c The miR-152-3p expression in patients with and without cancer family history. In both figures, LFC was used to show the expression level was normalized to U6 snRNA in the two groups. LFC means base the logarithm2 of fold change. 3. The correlation of miR-185-5p expression level with subgroup analysis. a The miR-185-5p expression in patients with and without abortion history. b The expression of miR-185-5p in patients ≤50 and >50 ages. c The miR-185-5p expression in patients with and without cancer family history. In both figures, LFC was used to show the expression level normalized to U6 snRNA in the two groups. LFC means base the logarithm2 of fold change. 4. The miR-152 target interaction depicted by Cytoscape 3.7. 5. Top 10 enriched biological processes among target genes of miR-152-3p. GO Gene Ontology, FDR false discovery rate. [file 13104_2021_5647_MOESM1_ESM.pptx]

## Slide 1
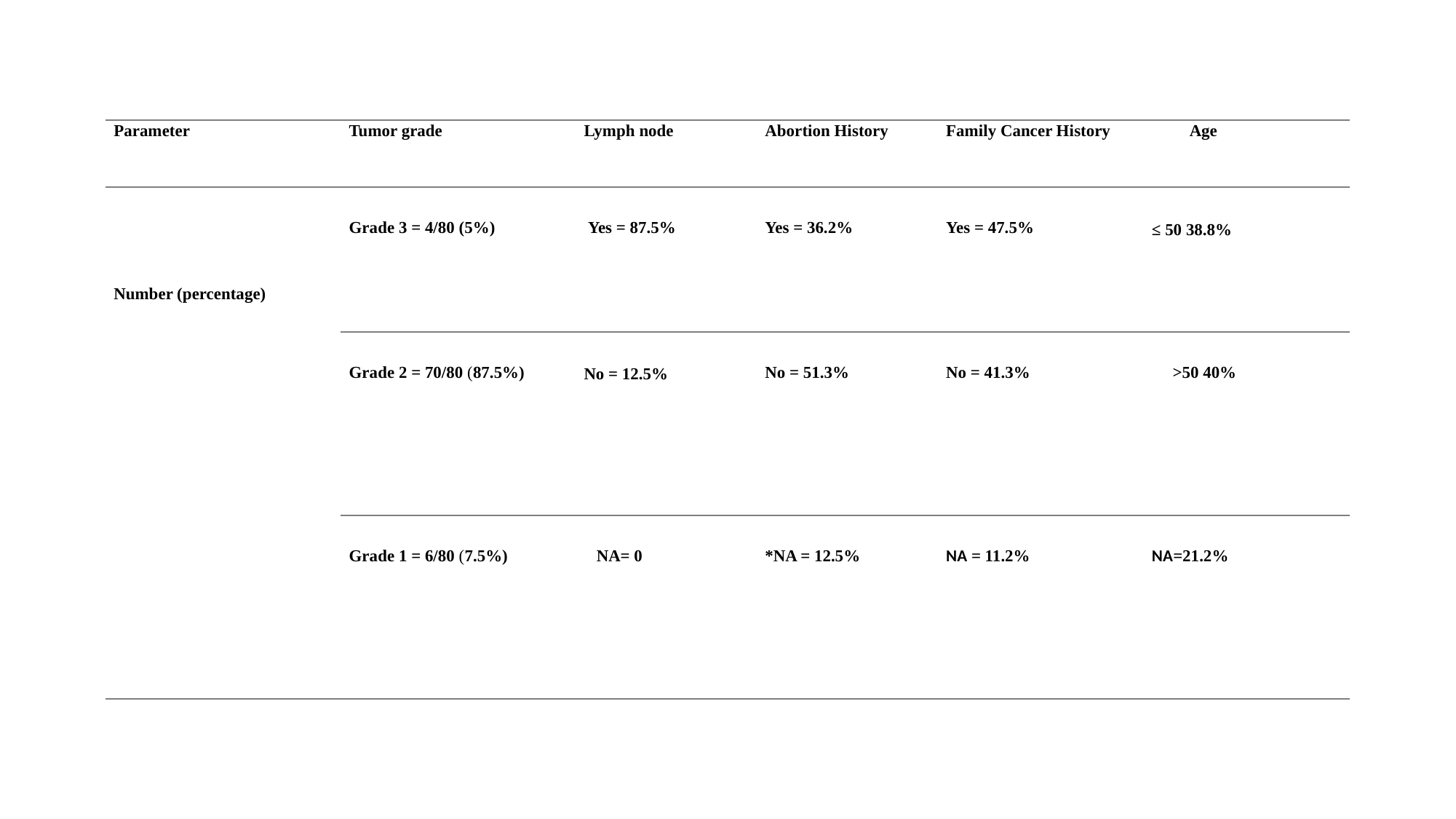

| Parameter | Tumor grade | Lymph node | Abortion History | Family Cancer History | Age |
| --- | --- | --- | --- | --- | --- |
| Number (percentage) | Grade 3 = 4/80 (5%) | Yes = 87.5% | Yes = 36.2% | Yes = 47.5% | ≤ 50 38.8% |
| | Grade 2 = 70/80 (87.5%) | No = 12.5% | No = 51.3% | No = 41.3% | >50 40% |
| | Grade 1 = 6/80 (7.5%) | NA= 0 | \*NA = 12.5% | NA = 11.2% | NA=21.2% |

## Slide 2
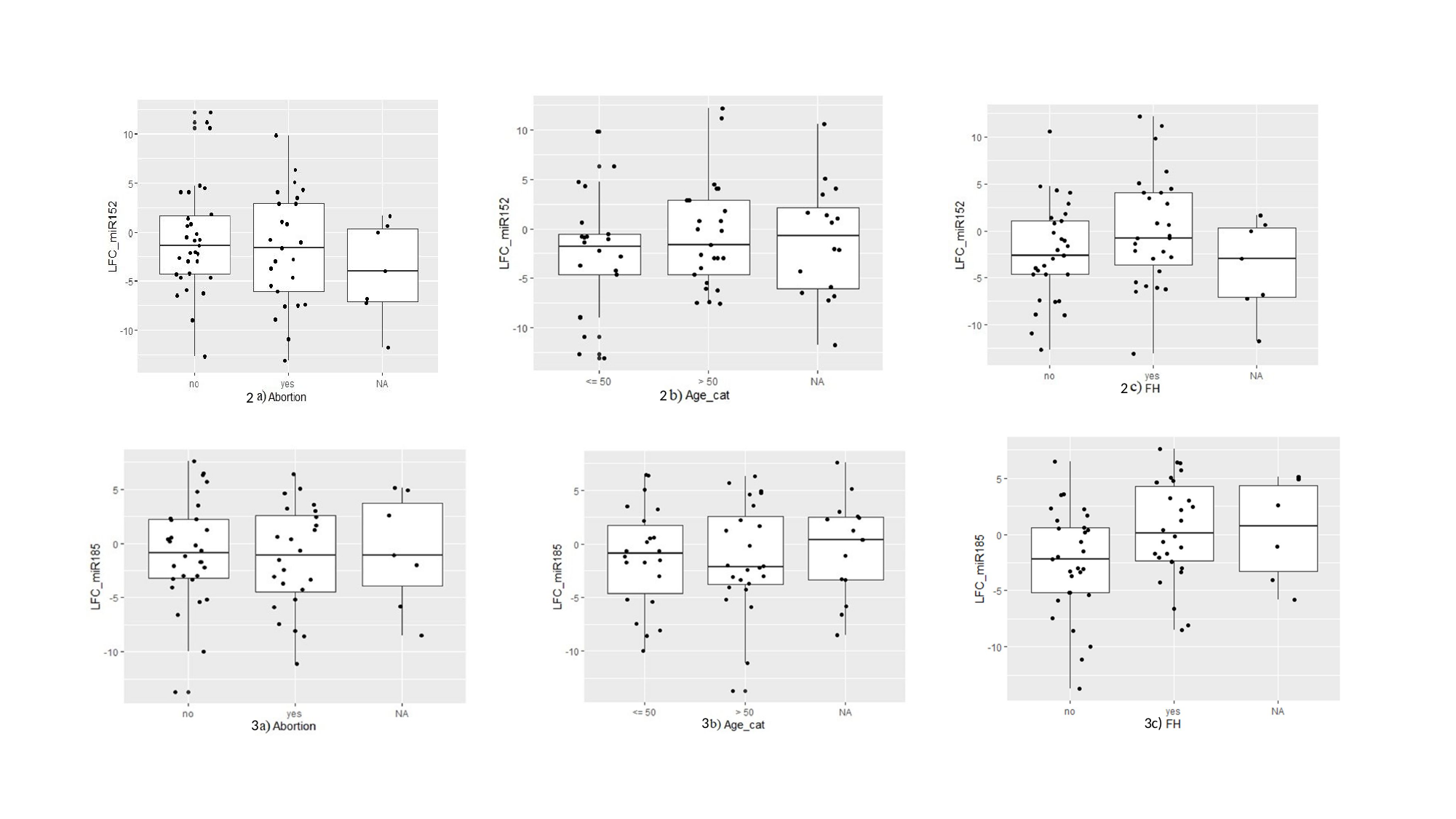

2
2
2
3
3
c)
3

## Slide 3
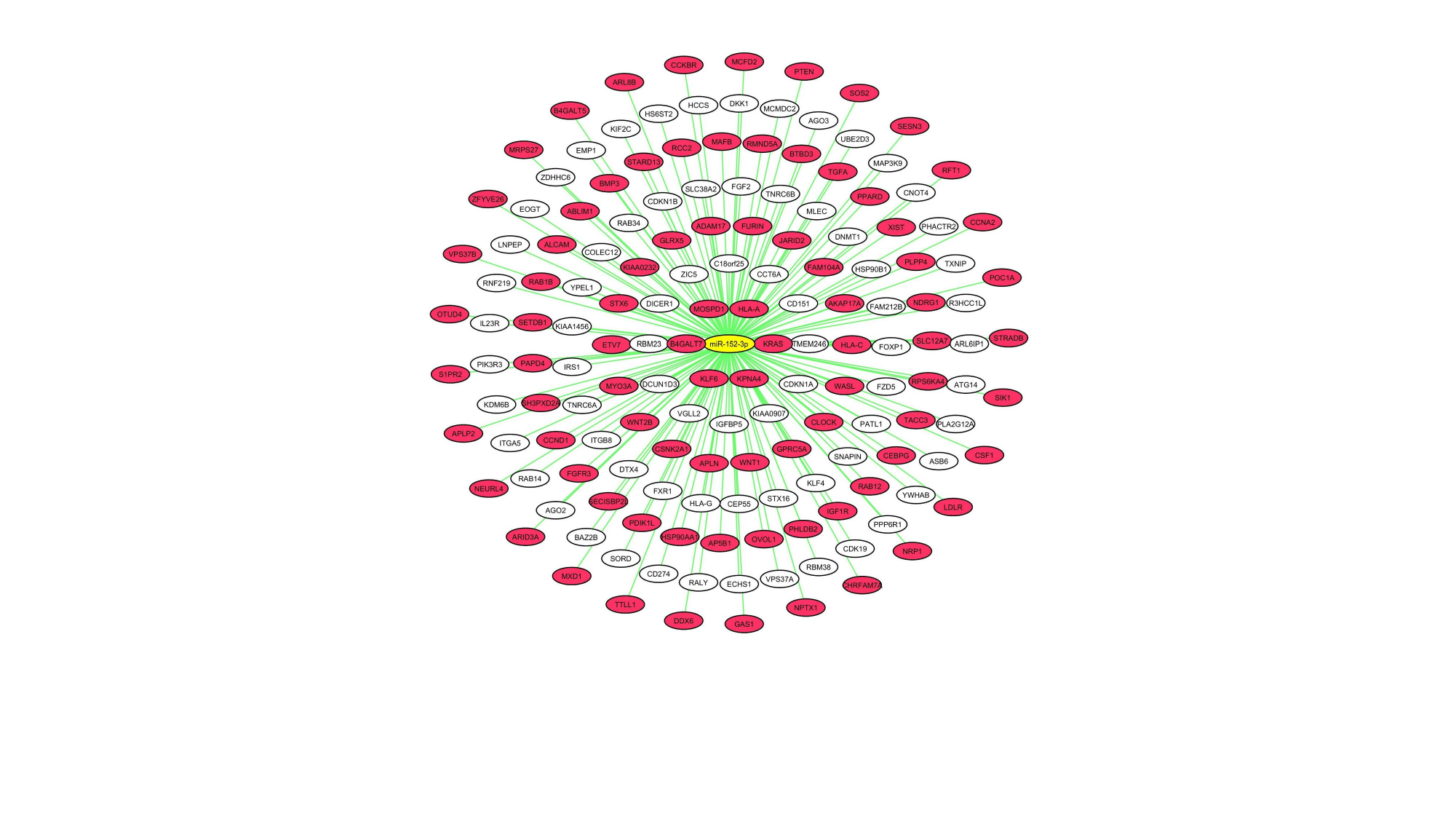

#

## Slide 4
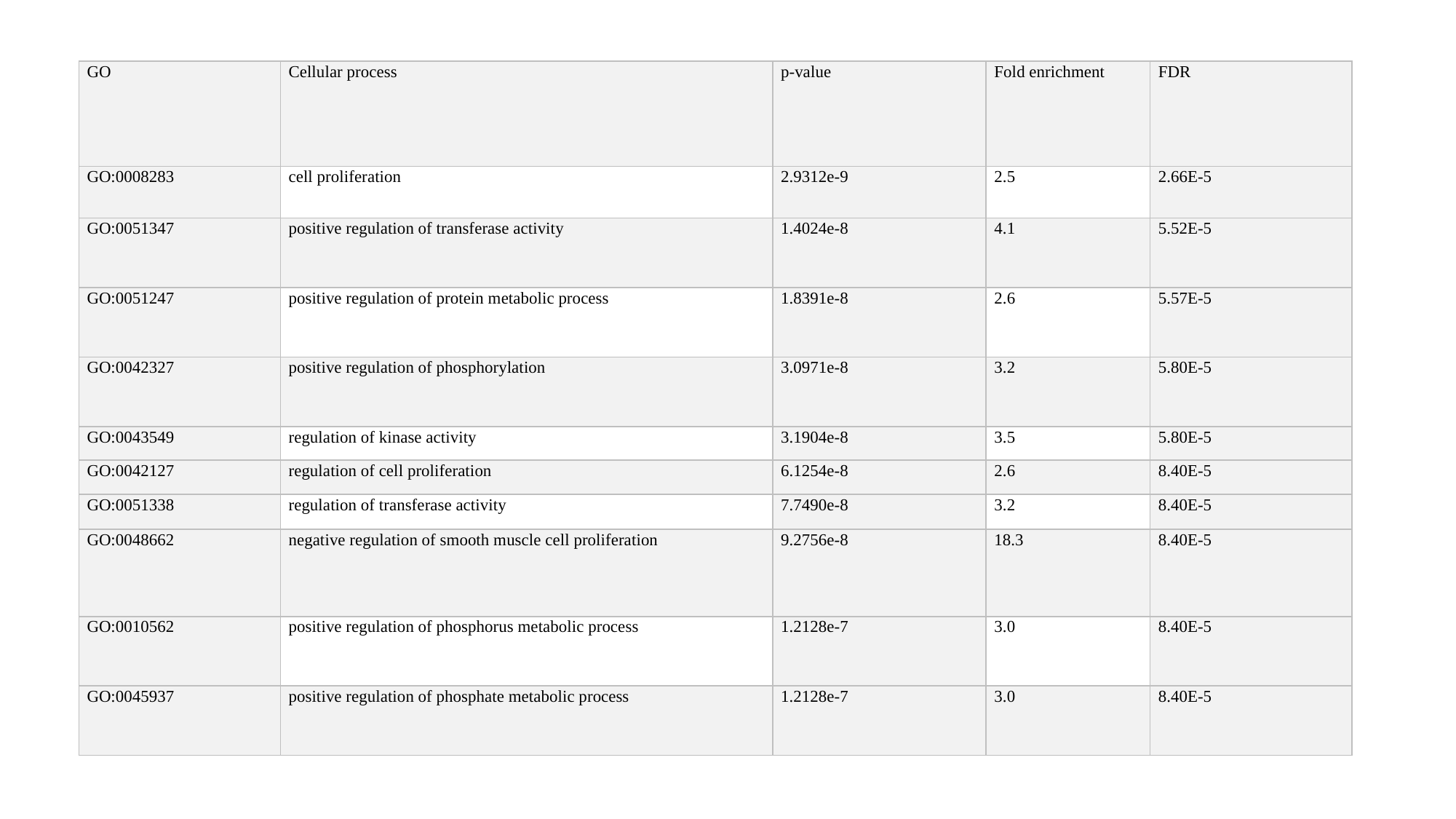

| GO | Cellular process | p-value | Fold enrichment | FDR |
| --- | --- | --- | --- | --- |
| GO:0008283 | cell proliferation | 2.9312e-9 | 2.5 | 2.66E-5 |
| GO:0051347 | positive regulation of transferase activity | 1.4024e-8 | 4.1 | 5.52E-5 |
| GO:0051247 | positive regulation of protein metabolic process | 1.8391e-8 | 2.6 | 5.57E-5 |
| GO:0042327 | positive regulation of phosphorylation | 3.0971e-8 | 3.2 | 5.80E-5 |
| GO:0043549 | regulation of kinase activity | 3.1904e-8 | 3.5 | 5.80E-5 |
| GO:0042127 | regulation of cell proliferation | 6.1254e-8 | 2.6 | 8.40E-5 |
| GO:0051338 | regulation of transferase activity | 7.7490e-8 | 3.2 | 8.40E-5 |
| GO:0048662 | negative regulation of smooth muscle cell proliferation | 9.2756e-8 | 18.3 | 8.40E-5 |
| GO:0010562 | positive regulation of phosphorus metabolic process | 1.2128e-7 | 3.0 | 8.40E-5 |
| GO:0045937 | positive regulation of phosphate metabolic process | 1.2128e-7 | 3.0 | 8.40E-5 |
